# Human-centered implementation research: a new approach to develop and evaluate implementation strategies for strengthening referral networks for hypertension in western Kenya

**DOI:** 10.1186/s12913-021-06930-2

**Published:** 2021-09-03

**Authors:** Mc Kinsey M. Pillsbury, Eunice Mwangi, Josephine Andesia, Benson Njuguna, Gerald S. Bloomfield, Agneta Chepchumba, Jemima Kamano, Tim Mercer, Juliet Miheso, Sonak D. Pastakia, Shravani Pathak, Aarti Thakkar, Violet Naanyu, Constantine Akwanalo, Rajesh Vedanthan

**Affiliations:** 1 Department of Medicine, University of California, San Francisco, San Francisco, CA USA; 2 Academic Model Providing Access to Healthcare (AMPATH), Eldoret, Kenya; 3 Moi Teaching and Referral Hospital, Eldoret, Kenya; 4 Duke University School of Medicine, Durham, NC USA; 5 College of Health Sciences, School of Medicine, Moi University, Eldoret, Kenya; 6 Department of Population Health, The University of Texas at Austin Dell Medical School, Austin, TX USA; 7 Center for Health Equity and Innovation, Purdue University, West Lafayette, IN USA; 8 Icahn School of Medicine, Mount Sinai, New York, NY USA; 9 Department of Population Health, NYU Grossman School of Medicine, 180 Madison Avenue, 8th floor, New York, NY 10016 USA

**Keywords:** Human-centered design, Implementation research, Hypertension, Referral networks, Peer interventions, Health information technology

## Abstract

**Background:**

Human-centered design (HCD) is an increasingly recognized approach for engaging stakeholders and developing contextually appropriate health interventions. As a component of the ongoing STRENGTHS study (Strengthening Referral Networks for Management of Hypertension Across the Health System), we report on the process and outcomes of utilizing HCD to develop the implementation strategy prior to a cluster-randomized controlled trial.

**Methods:**

We organized a design team of 15 local stakeholders to participate in an HCD process to develop implementation strategies. We tested prototypes for acceptability, appropriateness, and feasibility through focus group discussions (FGDs) with various community stakeholder groups and a pilot study among patients with hypertension. FGD transcripts underwent content analysis, and pilot study data were analyzed for referral completion and reported barriers to referral. Based on this community feedback, the design team iteratively updated the implementation strategy. During each round of updates, the design team reflected on their experience through FGDs and a Likert-scale survey.

**Results:**

The design team developed an implementation strategy consisting of a combined peer navigator and a health information technology (HIT) package. Overall, community participants felt that the strategy was acceptable, appropriate, and feasible. During the pilot study, 93% of referrals were completed. FGD participants felt that the implementation strategy facilitated referral completion through active peer engagement; enhanced communication between clinicians, patients, and health administrators; and integrated referral data into clinical records. Challenges included referral barriers that were not directly addressed by the strategy (e.g. transportation costs) and implementation of the HIT package across multiple health record systems. The design team reflected that all members contributed significantly to the design process, but emphasized the need for more transparency in how input from study investigators was incorporated into design team discussions.

**Conclusions:**

The adaptive process of co-creation, prototyping, community feedback, and iterative redesign aligned our implementation strategy with community stakeholder priorities. We propose a new framework of human-centered implementation research that promotes collaboration between community stakeholders, study investigators, and the design team to develop, implement, and evaluate HCD products for implementation research. Our experience provides a feasible and replicable approach for implementation research in other settings.

**Trial registration:**

Clinicaltrials.gov, NCT02501746, registration date: July 17, 2015,

**Supplementary Information:**

The online version contains supplementary material available at 10.1186/s12913-021-06930-2.

## Background

Hypertension is the leading preventable cause of early death and disability globally, and 75% of patients with hypertension live in low- and middle-income countries (LMICs) [1–3]. Notably, hypertension treatment and control rates in LMICs are very low. In Kenya, a comprehensive assessment of the hypertension care cascade revealed overall treatment and control rates of less than 10% [4]. Referral non-adherence and delays in referral completion are major barriers to chronic disease management and may be attributed to patient-related (cost, time required, transportation, low prioritization), provider-related (poor documentation, limited resources), and systems-level (lack of integrated electronic medical record, prolonged waiting times, transferring between health facilities) factors [5–7]. Conversely, stronger referral networks and referral strategies have the potential to improve health outcomes [8, 9]. In Kenya, the prevalence of hypertension has reached nearly 25%, and the Ministry of Health has targeted referral system gaps within a broader effort to improve hypertension control and access to care [10, 11].

Addressing gaps in the referral system requires a deep understanding of referral barriers (financial, logistical, and infrastructural) as well as the behaviors influencing referral completion. Potential solutions must prioritize the needs of community members, patients with hypertension, and healthcare providers who encounter these problems at the ground level. Human-centered design (HCD) is one method of promoting engagement of these key stakeholders to enrich understanding of local factors and create contextually-specific solutions. HCD offers a systematic approach to developing products and processes that center on the experience and core needs of the end-users [12]. This approach has been successfully adapted to design health interventions in LMICs, bridging the “knowing-doing gap” to pragmatically translate evidence-based approaches into improved health behaviors and chronic disease outcomes [13–15].

Recognizing that contextually-appropriate approaches are necessary in order to ensure high referral completion rates and ultimately improve blood pressure control, our team adopted an HCD process to develop the implementation strategy for the ongoing STRENGTHS study (Strengthening Referral Networks for Management of Hypertension Across the Health System). The STRENGTHS project ultimately aims to improve referral networks and coordination of hypertension care using a combination of peer-based support and health information technology (HIT) [16]. HCD is increasingly applied to implementation research, and we have successfully used this approach in previous studies [17, 18]. Here, we report on the process of developing and evaluating the STRENGTHS implementation strategy in western Kenya through an HCD approach prior to implementation in a cluster-randomized controlled trial.

## Methods

### Study setting

The STRENGTHS study focuses on improving referral networks for hypertension care across the public sector health system in western Kenya. STRENGTHS is embedded within the AMPATH (Academic Model Providing Access to Healthcare) program, a global health partnership between Moi University College of Health Sciences, Moi Teaching and Referral Hospital, and a consortium of North American academic medical centers led by Indiana University [19]. In collaboration with the Kenyan Ministry of Health, AMPATH has established a Chronic Disease Management (CDM) Program, which has enrolled over 40,000 patients with hypertension at over 70 health facilities spanning all levels of the health system [20, 21]. The multicomponent CDM care delivery package includes task redistribution [22], clinical decision support using HIT [23], consistent and secure medication supply [24], linkage and retention activities [25], community and stakeholder engagement [26], and social support for patients.

The CDM program has also used the AMPATH Medical Record System (AMRS), a customized version electronic health record to document and access patients’ clinical information across the health system [27]. The most updated version of AMRS, implemented in 2016, includes a software program called “Point-of-Care” (POC), which supports historical patient data review, real-time clinical data entry, decision support tailored to the type and training of the clinician, health facility management), and data visualization (e.g. quality indicators). The POC platform also includes an electronic referral form, in which referring providers document the reason for referral and other clinical information. This program is supported through a solar-powered Wi-Fi network to ensure network connectivity for each health facility. Where this infrastructure is not available, clinical data entry occurs retroactively through two alternative means: an offline tablet-based program (called mUzima) or through standardized paper forms, which are subsequently synced or manually entered, respectively, into AMRS. Regardless of the method of data capture, clinical data from all facilities is housed within AMRS.

### Conceptual approach

**Fig. 1:**
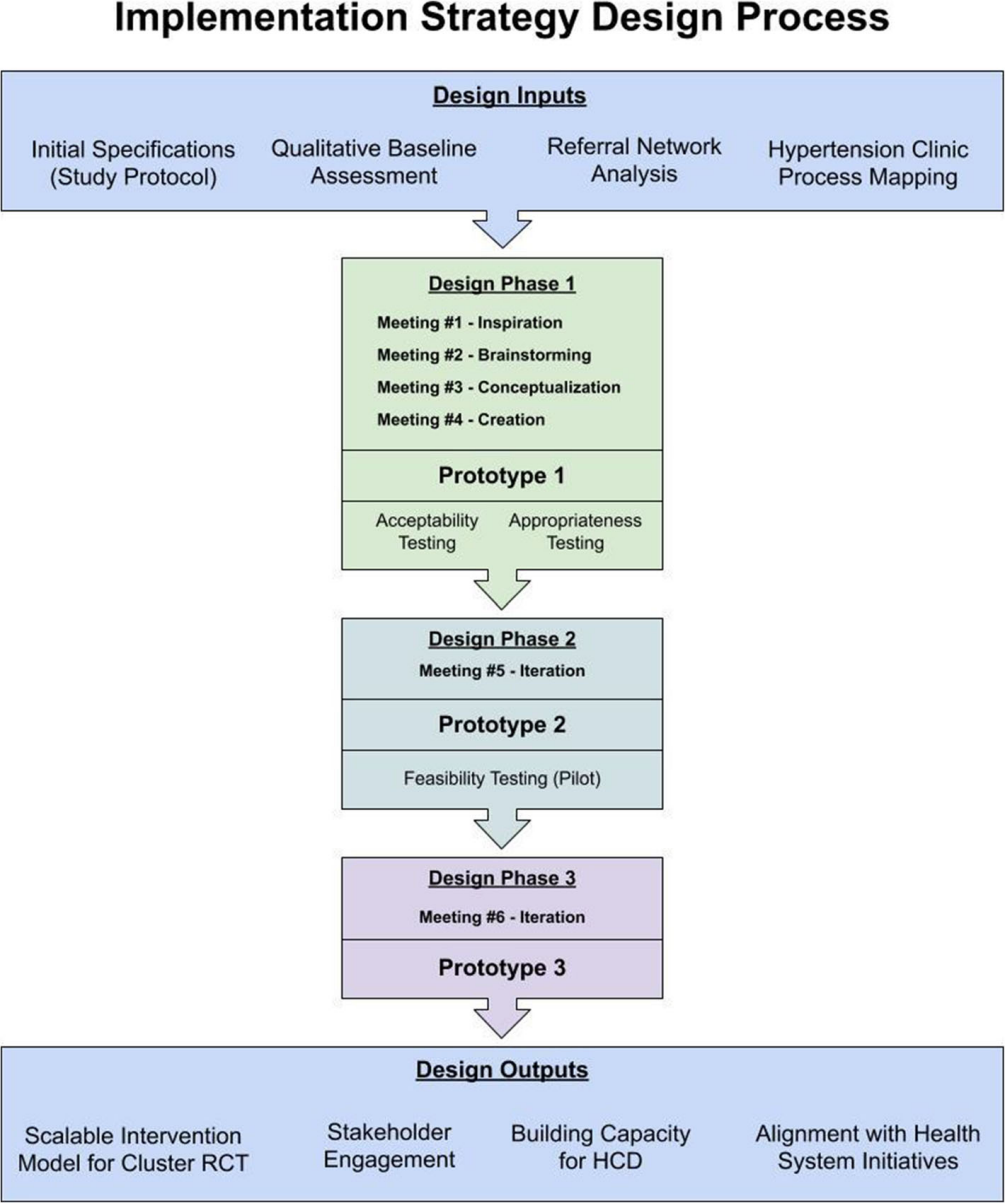
Participatory design process for the STRENGTHS implementation strategy

The HCD process was adapted from the Ideo.org Design Kit [28]. Our primary goal was to create an implementation strategy combining peer support and informatics that would meet the needs of end-users—patients referred for hypertension and clinicians. In addition, we aimed to engage community and health system stakeholders throughout the process. We organized the design process into three phases from January to September 2019 (Fig. 1). During design phase 1, we held four half-day design sessions to review preliminary findings from the baseline needs and contextual assessment and construct prototype 1, followed by acceptability and appropriateness testing. Then in design phase 2, we held a full-day design session to incorporate community feedback into an updated strategy (prototype 2), followed by the pilot study and feasibility testing. Finally, in design phase 3, we held a full-day design session to incorporate pilot feedback and finalize the implementation strategy (prototype 3). Between each design session, facilitators presented design progress to the study investigators, who provided additional feedback that was incorporated by the design team.

### Participants

The interdisciplinary design team consisted of 15 key stakeholders, including two patients with hypertension, two peer navigators from the HIV care program [29], three clinicians from primary- and secondary-level facilities, three health system administrators, three informatics experts, and two members of the STRENGTHS research team. Participants were identified through purposive sampling based on personal or professional experiences with hypertension referrals, peer support strategies, or clinical informatics. Preliminary data from referral network analysis, conducted during an early phase of the STRENGTHS study, further identified key clinicians who linked multiple parts of the referral system for inclusion on the design team [30]. All design team members represented end-users who may implement, receive, or administer the implementation strategy. The team was led by two facilitators from the STRENGTHS research team who were trained in HCD methods (JA, MP).

### Design inputs

Prior to design phase 1, the STRENGTHS research team conducted a baseline needs and contextual assessment that included qualitative evaluation of key barriers and facilitators to hypertension referrals, referral network analysis, and observational process mapping of hypertension clinic workflows [30, 31]. These data were summarized as key “insight statements” to align with HCD methods and presented to the design team. In addition, study investigators proposed initial design specifications for the implementation strategy, which included the use of peer support strategies and HIT to enhance referral adherence and patient tracking [16]. Initial specifications functioned as a scaffold that could be adapted and enriched through the design process through addition of contextually specific details (Fig. 1).

### Design phase 1

In design phase 1, the design team synthesized formative findings from the baseline needs and contextual assessment, reviewed initial specifications for the implementation strategy, and created prototype 1. During Meeting #1 (Inspiration), the design team grouped key insight statements into themes. During Meeting #2 (Brainstorming), the design team crafted “How Might We…?” questions to address each theme and brainstormed solutions. During Meeting #3 (Conceptualization), the design team grouped similar ideas together and refined solution ideas. During Meeting #4 (Creation), the design team selected the best solution ideas and integrated these into prototype 1 for community testing, comprised of detailed diagrams and storyboards depicting how stakeholders would interact with the implementation strategy.

Qualitative feedback on prototype 1 was gathered to assess acceptability (perception that the strategy is “agreeable, palatable, or satisfactory”) and appropriateness (perception of strategy “fit, relevance, or compatibility” to address hypertension referrals in our setting) [32]. Focus group discussions (FGDs) were conducted in English and Swahili with patients (*n* = 14), clinicians, and administrators (*n* = 15) at multiple health facilities. Participants were recruited through convenience sampling and provided written informed consent. The initial prototype diagrams conveyed key features of the implementation strategy to FGD participants and depicted use from the patient, clinician, and peer navigator perspectives. Subsequently, a structured discussion guide was developed to ensure data collected was relevant (Supplementary Files 1 and 2). The FGD sessions were moderated by a trained research assistant using the FGD structured guide to facilitate discussion and feedback among FGD participants. FGDs were audio-recorded, transcribed, and translated into English by research assistants. Transcripts underwent content analysis using Nvivo software. An a priori coding framework was established based on the discussion guide, and additional inductive codes were subsequently added. Emergent themes were identified which captured key strengths and challenges related to acceptability and appropriateness. All participants were provided a transport allowance and refreshments.

### Design phase 2

During Meeting #5, the design team incorporated feedback from community FGDs and created prototype 2. Following this session, we conducted a pilot study to test prototype 2 for feasibility (perception that the strategy can be “successfully used or carried out within a given agency or setting”) [32]. We selected three pilot clinics, one at each level of the health system, thus constituting a referral chain. Three peer navigators were recruited from the study sites, trained in core roles and responsibilities, and equipped with the HIT package via tablet. Peer navigators were hired members of the clinical team and were compensated for their work. Each peer was stationed at a specific clinic and was able to communicate directly with the peer stationed immediately above or below in the referral chain. We recruited 15 patients referred among these three facilities over a four-month period. Inclusion criteria included age greater than 18 years and currently enrolled in the AMPATH CDM program; exclusion criteria included acute illness requiring immediate medical attention, terminal illness, or inability to provide informed consent. Patient referral encounters were analyzed for referral completion, reason for referral, and reported barriers to referral.

We subsequently assessed feasibility of prototype 2 via FGDs with patients (*n* = 12), clinicians, and administrators (*n* = 13) who participated in the pilot, as well as semi-structured interviews (SSIs) with each peer navigator (*n* = 4) (question guides available in Supplementary Files 1, 2 and 3). These discussions were recorded, transcribed, translated, and underwent content analysis in the method described above in order to capture key strengths and challenges related to feasibility.

### Design phase 3

During Meeting #6, the Design Team incorporated the FGD and SSI results in order to enhance feasibility, yielding prototype 3. This final model was then implemented in the STRENGTHS cluster-randomized controlled trial.

### Design team reflections

In order to capture reflections on the HCD process, design team members (*n* = 14) participated in FGDs after Meeting #5 (Design Phase 2) and completed a 32-item 6-point Likert-scale questionnaire after Meeting #6 (Design Phase 3). FGDs were conducted in English using a structured discussion guide and were audio-recorded, transcribed, and analyzed for content in the method described above. The Likert-scale questionnaire was adapted from the Community-Based Participatory Research community engagement survey and included the following four domains: contextual factors, partnership processes, research method perceptions, and participatory outcomes [33, 34]. The questionnaire was administered individually to each design team member by a research assistant using a tablet-based RedCap tool.

## Results

### Design phase 1

**Fig. 2:**
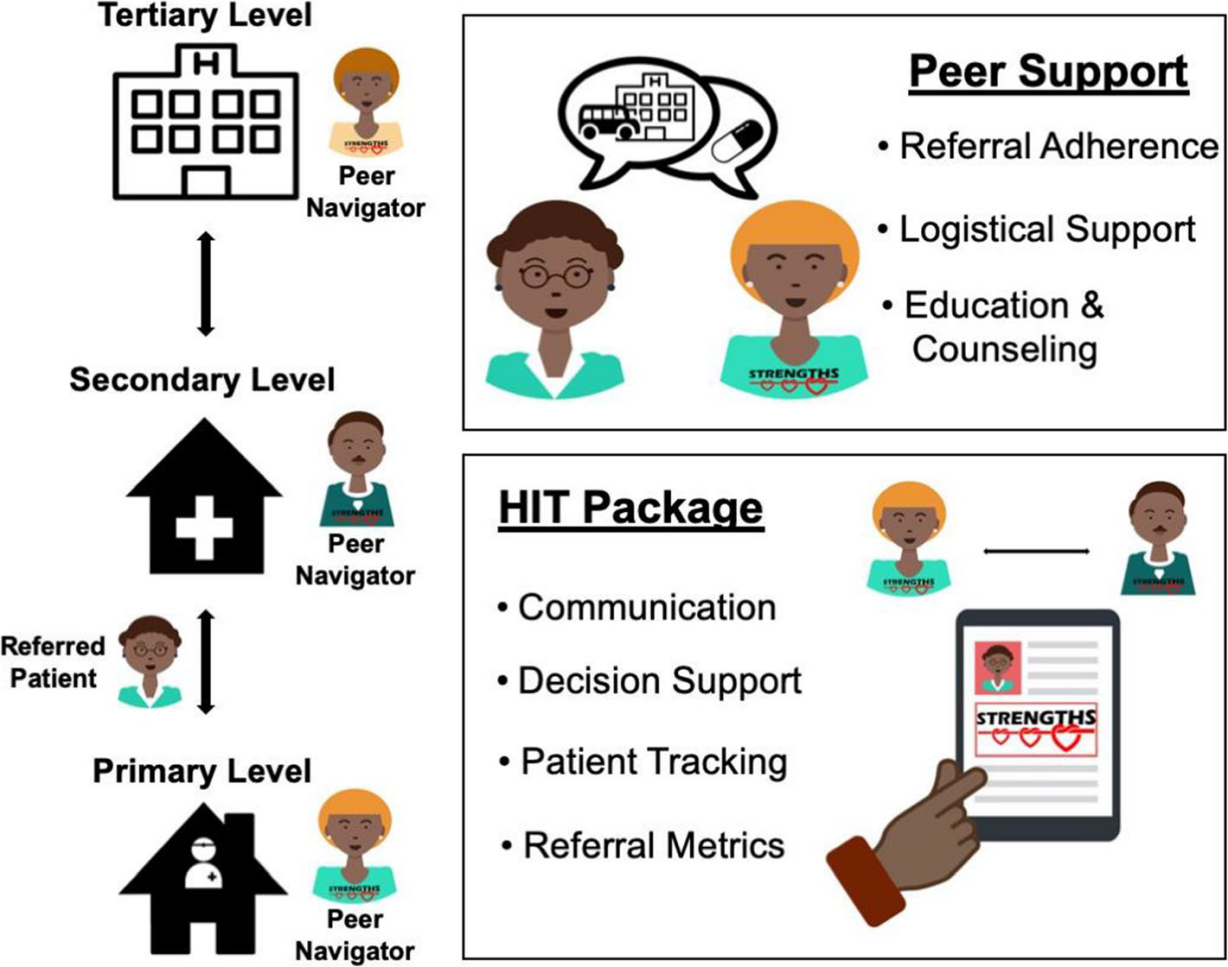
The STRENGTHS Implementation Strategy (Prototype 3) - Peer navigators meet with referred patients at to facilitate referral completion. Peer support strategies include referral adherence, logistical support, education, and counseling. The health information technology (HIT) package captures patient movement in AMRS and enhances communication between clinicians, decision support, patient tracking, and monitoring of key referral metrics

The initial implementation strategy (prototype 1) consisted of a peer navigator program and HIT package for patient tracking, documentation, and decision support (Fig. 2).

#### Peer navigator program

Peer navigators were patients with hypertension who had achieved blood pressure control and/or successfully completed inter-facility referrals, and they were hired as members of the clinical team and compensated for their work. All peer navigators underwent standard training on patient privacy and handling of protected health information. These peer navigators would meet with referred patients at both the referring and receiving facility in order to facilitate referral completion. Core responsibilities of the peer navigator included logistical navigation, education, and psychosocial support. Logistical navigation ensured that patients knew the correct date, time, and location of the next clinic, estimated costs associated with the referral visit, mode of transport, and what to expect during the referral appointment. Education included reviewing basic facts about hypertension and the patient’s specific reason for referral. Psychosocial support leveraged the peer navigator’s shared disease experience of hypertension to emphasize the importance of referral adherence.

#### HIT package

Patient movement between facilities was captured in the HIT package, which facilitated transmission of information among all peer navigators, clinicians, and administrators involved in a referred patient’s care. Based on the foundation of AMRS and existing electronic clinical encounter forms described above, we programmed additional functionalities for peer navigators and referring clinicians. This included a “peer navigator encounter form” to document patient counseling sessions and supplement clinician referral forms, which included the reason for referral and other relevant clinical information for the next provider. In addition, the HIT package included integrated decision support to guide peer navigator discussion topics and basic clinical actions.

#### Acceptability and appropriateness testing

Overall, prototype 1 was found to be acceptable and appropriate to patients, clinicians, and administrators. In addition, participants identified several specific prototype strengths and challenges to be addressed through the next design phase (Table 1).

**Table 1:** Acceptability & Appropriateness Testing by Stakeholder Group

	**Acceptability**		
	**Theme**	**Patients**	**Clinicians/Administrators**
**Peer Support**	Improved Patient Experience	Peer navigators may address inefficiencies and delays during referrals	Peer navigators will help patients navigate unfamiliar health facilities
	Stigma	Peer navigators may reduce perceived bias and judgement by clinic staff through accompanying patients and orienting them to the new health facility	Patients may feel uncomfortable disclosing personal health information to other community members (Peer navigators, who are not seen as health professionals)
	Integrating Peer Navigators into local clinics	Due to regional differences in culture and language, patients referred to a different facility (in a different community) may not feel comfortable or trust their receiving peer	Nurses and administrators currently help facilitate referrals, and there may be conflict when peers come in to assume this role
**HIT Package**	Information Sharing Between Facilities	Referral information (including clinical data) can be available to clinicians at facilities across the health system	Electronic forms must be streamlined and user-friendly, as clinicians have encountered inefficient forms in the past
	HIT Reliability	(No patients commented on HIT reliability)	The intervention would need a backup so that data is not lost and the core functionality of referral navigation may proceed in the event of power or network outage
	**Appropriateness**		
	**Theme**	**Patients**	**Clinicians/Administrators**
**Peer Support**	Capacity of Peer-based Education	Peer navigators may provide patient education on hypertension on multiple occasions during referral process	Patients with hypertension may be inappropriate peer support providers as they may be older, harder to retain, and less familiar with technology; in addition, these patients lack formal health training necessary to provide peer support (participants advocated for use of Community Health Volunteers
	Prohibitive Costs	Without transport or funds, referral barriers may still be insurmountable for patients	Patients may not complete referrals without providing transport, incentives, and/or peer accompaniment
	Peer Navigator Accessibility	Peer navigators may be inaccessible or unreachable when needed by patients	Concerns that peer navigators may be difficult for patients to locate at busy facilities like MTRH
	Peer-Clinician Information Sharing	(No patient comments on appropriateness of Peer-Clinician Interactions)	Peer navigators will effectively relay clinical information between clinicians at different facilities during referrals
**HIT Package**	Centralized Data Storage	(No patient comments on appropriateness of HIT tools)	Referral data is stored centrally and can be accessed via tablet by referring or receiving clinicians
	Integration Barriers with Existing Record System		Clinical information must be accessible no matter what health record system is used, meaning that the intervention must integrate all existing systems

Patients felt that the STRENGTHS implementation strategy would improve their experience of the referral process and facilitate referral completion. Patients felt that the proposed approach would improve logistical experience during referrals, reduce stigma through peer support and advocacy, strengthen patient education, and facilitate information sharing between facilities. Clinicians and administrators similarly felt that the STRENGTHS implementation strategy would augment their abilities to refer patients and facilitate referral adherence through improved communication and health data access.

Patients also cited several concerns with the strategy and identified three main challenges: referral-related costs (e.g. transport, healthcare fees), persistent cultural and language barriers between patients and peer navigators, and lack of peer navigator accessibility. Clinicians and administrators also shared the concern that the implementation strategy did not address many barriers to referral completion. They felt that without organized transport, stipends, or other incentives, patients would be unable to complete referrals. Clinicians and peer navigators were also skeptical of peer navigators’ level of knowledge or training and ability to integrate into the clinic setting.

While clinicians and administrators generally liked the idea of using technology to facilitate referrals, they also emphasized the practical challenges of technology in the rural setting. Frequent network or power outages at rural clinics could interfere with tablet charging or internet use. They emphasized the need for paper back-up forms and poor usability of prior electronic encounter forms. Finally, clinicians and administrators raised concerns that the HIT package may be unable to coordinate between clinics using different health record systems (especially those with paper-based record systems).

### Design phase 2

#### Design adaptations

The design team incorporated feedback from acceptability and appropriateness testing to create prototype 2. To address the patient concerns, we equipped peer navigators with airtime credits to ensure reachability via phone and stationed them at the health facility during clinic hours; additionally, we recruited peer navigators from local communities near each facility to strengthen the sociocultural bond with patients. To address the clinician and administrator concerns, we expanded peer navigator training to augment medical knowledge and communication skills, coordinated development of the HIT package to align referral tools with the appearance, workflows, and features of AMRS, and organized training sessions for clinicians and administrators on hypertension referral algorithms and use of AMRS. Prototype 2 was subsequently implemented in three hypertension clinics and tested for feasibility.

#### Feasibility testing

Seventeen patients referred for hypertension care were screened and 15 of these (4 male, 11 female) met inclusion criteria and enrolled in the pilot study. All patients were referred up to higher levels of care; there were no down-referrals. Seven patients were referred from the primary level and eight from the secondary level. Fourteen pilot participants (93%) completed the referral. The one participant who did not complete the referral was referred for investigations (diagnostic tests) and met with a peer navigator, but after consulting with her family decided against attending the appointment. Reported referral barriers included costs of attending the referral (40%), transport logistics (7%), and insurance coverage (7%). Despite the stated barriers, these patients successfully completed the referral. Eight patients (53%) reported no barriers to referral completion. Pilot feedback supported the feasibility of prototype 2 in hypertension clinics. Thematic analysis identified multiple perceived strengths and challenges (Table 2) aligned with 3 central domains: active peer engagement, enhanced communication, and referral data integration.

**Table 2:** Feasibility testing results, summarized by themes and representative quotations

Feasibility Testing			
Domain	Key Theme		Quotation
**Active Peer Engagement**	Effective Patient Navigation	Peers provided logistical support and oriented patients to unfamiliar health facilities	*Someone like me, if am told to go to Referral [Moi Teaching and Referral Hospital], where would I start from to get to the doctor? Because in Referral people are many and you can’t even know which room you will go to so that you be served immediately. [I] am thankful, that program [STRENGTHS] is good. (Patient, Turbo Secondary Level Facility)*
	Improved Patient Advocacy	Patients felt comfortable talking with peer navigators, who functioned as patient advocates and often accompanied patients into their clinician visits	*The patient is more free to explain himself to the peer navigator whom he seems to see as a normal person…when the patients knows that he is going to talk to the Doctor, he thinks to prepare him so much that sometimes he ends up forgetting other things but when he is talking to the peer navigator, he feels very free…but when with the Doctor, the patient may come out and say I had forgotten to tell the provider A, B, C, D. And so mostly the patient feels comfortable with the peer navigators when they are explaining their thoughts. (peer navigator, Moi Teaching and Referral Hospital)*
	Active Peer Follow-up	Active follow up from peer “challenged” patients to overcome barriers that may have prevented them from completing referrals	*I am grateful because [the Peer Navigator] will even challenge you. If you have lacked money you will run to the neighbor and say, ‘there is someone somewhere who wants to help me.’ So I run and found the transport and I went.* (Patient, Turbo Secondary Level Facility).
	Prohibitive Costs	Remaining challenges of referral completion included high cost of transport, drugs, and tests	*Sometimes money becomes a challenge. I went [to the hospital] and [the] doctor wanted to do some tests...I was told one is 1800 KSH and the other is 900 KSH and I didn’t have that money. I had to go back home. (Patient, Cheramei Primary Level Facility)*
	Limited Scope of Patient Education	Peers lacked training to counsel patients with multiple comorbidities or educate on topics other than HTN	*They say [my] eyes have been bad because of hypertension and because of diabetes. So mostly you are told [by peer navigators] that [they] have nothing to do on the side of the eyes. It’s better you go check on the eyes in Nairobi or where you will go. So what I can suggest is that diabetes and [high blood] pressure it affects the eyes, so they bring again here an optician so that he can direct us as well. (Patient, Turbo Secondary Level Facility)*
**Enhanced Communication**	Timely Referral Updates	Peers effectively communicated referral updates with clinicians and patients	*Communication was effective, was good communication. I think when you have a case to refer it was good to go to the desk where the peer is and the good thing she was always available, so we actually had no problem to go there. So, at times she can just come and explain this is a referral from the facility so we could give them a priority. So communication is very effective. (Clinician, Turbo Secondary Level Facility)*
	Improved Patient Understanding of Reason for Referral	Peers helped explain the reason for referral and convinced patient of the benefits of referral completion	*After being told from the doctor [that they must go to a referral facility], they end up not realizing really the exact meaning. But when we impact on them and explain more about why, in fact they end up realizing that we, they have understood the reason for referral.* (peer navigator, Moi Teaching and Referral Hospital)
	Convenient Access to Peers via Phone	Phone calls were most convenient means of planning and following up with patients	*[The peer navigator] took my phone number and I went home and she has been following me up from home...she calls me, she asks me how things are, even when I come here she asks me, “you have come, how are going on with your clinic?.” So they help so much. (Patient, Turbo Secondary Level Facility)*
	Successful Integration with Clinic Staff	Peers integrated well with clinic staff and assisted in other clinic tasks (e.g. triage vitals and patient education)	*We also engage the peer in providing health education to our clients and they also assisted us in working in other areas in the facility. (FGD, Providers, Turbo)*
	Reinforced Referral Practices	The presence of peers seemed to reinforce adherence to clinical protocols and increase clinicians’ awareness of referral resources	*After the providers understood the referral network, for the first weeks we didn’t have so many referrals but as time went by, they understood our role in the clinic…So, our communication was really good. Yes. They even come and look for me because am always at the front desk, they have a referral, the doctor comes to me and tells me I have such a referral. If I need them, I also go to their offices, I tell them what I need from them so, it was a two-way traffic.” (Peer Navigator, Turbo Secondary Level Facility)*
	Interruption of Clinic Workflow	Peers sometimes interfered with clinic flow if they spend too much time with the patient	*Patients have a priority to be seen by the consultant and then probably the consultant to the clerks and then to go home and then this peer educator comes in between. The patient is not ready for his or her service, so this navigator must be very convincing and actually bring the patient closer so that the patient can be able to give him or her time. (Clinician, Moi Teaching and Referral Hospital)*
	Difficulty Finding & Identifying Peers	Providers sometimes struggled to identify peer navigators in busy clinics without an established work station or uniform	*But my challenge has always been, how will this be a navigator be identified? (Clinician, Moi Teaching and Referral Hospital)*
**Referral Data Integration**	Fast Retrieval of Clinical Information	Use of a tablet enabled fast retrieval of referral information (when EMR available)	*From the word “go,” when a patient is referred we are able to know why the patient is referred from the [health record] system…it makes work easier. Yes. You don’t have to make phone calls [or] ask so many questions.* (peer navigator, Turbo Secondary Level Facility).
	Utility of Encounter Forms	Encounter forms provided some useful information that was referenced by other peers (even though most information sharing occurred over phone)	*[The HIT package] helped. You see, when the peer navigator was at [the] other facility [you] could click and see the updates about the patient. Then from there one could be able to know what is supposed to do next about the patient. (Peer Navigator, Moi Teaching and Referral Hospital)*
	Reliability of Network and HIT Package	Peer navigators had consistent access to the HIT package via a tablet and mobile data network	*For my case, there [was] no time [when I couldn’t use the electronic form] because...we used to have the [mobile data] bundles, which we were to buy and use it to fill the forms. So, I never [had problems with the network] but some other people experienced the same problems because the [clinic’s wifi] network was down. (peer navigator, Moi Teaching and Referral Hospital)*
	Lack of Physical Patient Reminders	Keeping track of paper referral forms, slips, or clinic booklets was challenging for patients	*If you are given that small card [referral appointment card] I can put in this bag. And tomorrow I want to go to the hospital [and] I have forget it was in another bag...now you find there is challenges because you will forget [the card]. (Patient, Cheramei Primary Level Facility)*
	Integration Barriers with Paper Record System	Some clinicians still used paper encounter forms due to simplicity, reliability, and availability (some clinics did not have access to EMR), which limited clinical information available in the EMR	*We used the written [paper] forms, up to now as am talking. The forms are in the POC [EHR], but finding them is a problem. But when you click you need to add “Sending to MTRH” in that form you are referring...now from Moi Teaching and Referral Hospital we need to add where specifically we need--is eye clinic, is renal unit--[the electronic form] doesn’t have that specification. Is it to oncology? You need to specify you are sending to oncology and a bit of notes what are you going to do. (FGD, Provider, Turbo)*
	Limited Provider Use of Referral Forms	Some clinicians were unaware of a standard referral form (instead used a freehand note), limiting referral information available in the EMR	*We use the internal consultation forms [for referrals]...But I have seen there are those [clinicians] who don’t want to use the consultation forms from MTRH and so we just write a letter which will be stamped by the hospital...But we don’t have a specific referral form for referring patients” (FGD, Provider, Turbo)*
	Limited Utility of HIT Package in Paper-based Clinics	It was difficult to use tablets in paper-based clinics (when the patient’s information was held in paper charts or not recorded in a standard way)	*The patient could just be written something on a paper [*e.g. *when clinicians did not use the standard referral form]. Yes. But no more information...You just get a written document that they have been referred for investigations.* (peer navigator, Turbo Secondary Level Facility)

##### Active peer engagement

Patients described how frequent contact with the peer navigator reduced stress and improved engagement in care. Improved referral navigation included both logistical planning before the appointment as well as orienting the patient upon arrival to the unfamiliar health facility. In addition, patients generally felt more comfortable discussing health topics with their peers and often requested that the peer navigator accompany them during the clinician visit as an advocate.

Participants voiced persistent concerns about transport and other referral-related costs, but emphasized how active follow-up from peer navigators helped patients to overcome these barriers. Peer navigators provided persistent appointment reminders, helped patients brainstorm ways to gather funds and leverage their social networks for support, and emphasized the benefits of engaging in referral care, including preventing disease complications and associated downstream healthcare costs. Patients and peer navigators described that these peer-based counseling strategies often enabled patients to attend referral appointments despite significant financial barriers.

##### Enhanced communication

Peer navigators enhanced communication between clinicians, administrators, and patients. Frequent follow-up (usually by phone) addressed any patient questions and ensured that patients understood the reason for referral. This filled a key communication gap between patients and clinicians, who had limited time for discussion during clinic visits. Peer navigators also provided referral updates to clinicians and alerted them when a new patient referred from an outside facility had arrived. This was especially important in clinics without AMRS access, in which peer navigators also provided relevant clinical context for the referral.

In addition, peer navigators reminded clinicians of the clinic referral pathways and the resources available to referred patients, ultimately improving provider referral practices. Peer navigators felt that the frequency of referrals increased over the course of the pilot as clinicians learned to collaborate with the peer navigators.

##### Referral data integration

The HIT package supported core peer navigator responsibilities, enabling fast retrieval of clinical information and documentation of referral information through tablet-based encounter forms. Peer navigators felt that the HIT package helped coordinate patient follow-up, reducing the need for peer-to-peer phone calls to convey patient information to one another. In addition, use of tablets with mobile data bundles ensured reliable AMRS access for peer navigators despite occasional power or clinic WIFI outages.

However, there were multiple challenges integrating with paper-based record systems. Prototype 2 was designed to be used with both paper charts and electronic data entry forms. When clinics used an electronic record system, peer navigators could easily retrieve relevant patient information. In contrast, clinicians in paper-based clinics rarely used a standardized form, making it difficult for peer navigators to interpret the reason for referral and clinical context.

This issue reflected larger challenges in expanding the implementation of POC throughout AMPATH clinics. As described by pilot clinicians, many clinics continued to use paper forms due to simplicity, reliability, and availability. Moreover, some clinicians used nonstandard referral forms (often a freehand note), resulting in little referral information available to other providers. In these instances, the peer navigator encounter form provided basic referral information in the electronic health record and ensured that some information was relayed to the next clinician. In summary, the HIT package was useful for peer navigators but faced significant integration barriers with the existing health record systems.

### Design phase 3

#### Design adaptations

The design team incorporated feedback from feasibility testing and created prototype 3 for implementation in the cluster-randomized controlled trial. To address concerns related to the patient referral experience, we further expanded peer navigator medical knowledge to include complications of uncontrolled hypertension and common medical comorbidities (e.g. diabetes mellitus). We strengthened peer navigator follow-up by developing a peer navigator-specific patient list on AMRS to highlight incomplete referrals. In response to the issue of averting potentially unnecessary referrals, we developed a workflow for the peer navigator to utilize. For example, if a patient was referred to a higher level facility for poorly controlled hypertension, and the peer navigator learns that the patient has been nonadherent to medications, then the peer is able to provide adherence counseling, discuss with the referring clinician, have the patient return to the referring clinician for further management and adherence counesling, and thereby effectively “cancel” the referral. Finally, to improve integration with clinic providers, we provided peer navigators with uniforms to facilitate identification and encouraged them to assist in clinic tasks during free time (e.g. triage vital signs).

Multiple modifications expanded the HIT package and improved integration with existing health record systems. We enhanced peer navigator decision support using branching logic embedded in encounter forms. We additionally created a dashboard platform for monitoring real-time referral metrics for each facility through AMRS, which was designed to ensure maintenance of fidelity and quality of the implementation strategy. Finally, the STRENGTHS research team liaised with CDM leadership to create a standardized referral form to be used by the referring clinician (available on paper and electronically) with standardized reasons for referral. These were introduced to each clinic through structured sensitization and training led by local leadership.

Overall, integration across multiple health record systems remained the biggest challenge to implementation. While some aspects of the HIT package offered a one-size-fits-all solution, the final prototype was optimized for clinics that used the electronic POC system. The CDM program is currently scaling up POC across the health system, but paper-based clinics will not benefit from the full functionality of the HIT package.

#### Design team reflections

Design team members reflected on their overall perceptions of the HCD process through FGDs and a survey following design phases 2 and 3, respectively (Table 3). The survey was administered to 14 participants (8 women and 6 men) with median age 39.5. Participants attended nearly all of the sessions (average 5.6 out of 6). FGD participants identified several strengths of the HCD process: participation from all design team members regardless of hierarchy, moderators’ ability to promote group consensus when conflicting opinions emerged, and representation of key stakeholder groups on the design team. Perceived challenges included: time management, refining solution ideas after prototyping, and promoting patient contributions during later stages of the design process. Participants emphasized the need for transparency and power-sharing in three major domains of the design process: 1) prioritizing session discussion topics; 2) selecting solution ideas for further development; and 3) incorporating feedback from the study investigators, who were external to the design team. Overall, design team members felt that they had made valuable contributions to the intervention and gained skills in communication, collaboration, and HCD methods through the design team experience.

**Table 3:** Design Team Survey Results

Design Team Reflections	Completely Agree or Mostly Agree
**Contexts**	
The STRENGTHS research team understands the needs of the community	100%
Some perspectives are NOT represented on the Design Team (Yes/No)	29%
	Other perspectives desired included: village leadership (chief, elders, church leaders), public health officers, policy makers
**Partnership Processes**	
Participation in discussion was equal among all DT members	100%
Discussion was dominated by a few individuals on the DT	7%
I felt that my opinions were taken into consideration during the design process	93%
**Intervention Design & Research**	
I liked how input from the STRENGTHS investigators was used to select solution ideas	64%
I felt that decision-making power was shared equally between the DT and STRENGTHS Research team	71%
**Participatory Outcomes**	
I felt that I was a valuable member of the DT	100%
I felt there were barriers or consequences to participating in the DT	7%
	Barriers included: travel time (distance), work conflicts, time required, difficulty of design work
Participation in the DT taught me skills that I will use in the future	100%
	Skills included: communication skills (listening, respectful discussion, building consensus), HCD methods (brainstorming, empathy, refining ideas, implementation), collaboration (especially with those with different perspectives), eliciting community feedback, how to navigate the health system, how to serve patients
At the end of the design process, I felt that the final STRENGTHS intervention reflected decisions made during Design Team meetings	100%
I trust that the STRENGTHS research team will implement the intervention as specified by the Design Team	71%
Constructive Feedback	More voting or rating of discussion topics
	Shorten the process so it can be applied to urgent issues
	Start sessions later to allow for participant transport
	Break up more difficult sessions over multiple days
	Involve more patients and have a component that directly educates them about HTN and referrals
	Use less medical language and jargon during sessions
	Involve patients and other stakeholders earlier in the design process so they can help draft the research proposal

## Discussion

We used an HCD approach to design the STRENGTHS implementation strategy, which overall was found to be acceptable, appropriate, and feasible for facilitating referrals for hypertension care. Participants recognized the strategy’s potential to improve referral completion and identified consistent strengths across multiple prototypes. Primary among these were referral facilitation through active peer engagement, enhanced communication between patients, clinicians, and administrators, and referral data integration through the HIT package.

Coordination with health system reform was critical to the success of our approach. For instance, we collaborated with the CDM program to update hypertension care protocols that included reasons for referral and referral pathways. During implementation of the pilot study portion of our project, peer navigators became integrated into clinic and collaborated closely with clinicians and staff. We observed that the frequency of referrals increased during this process, suggesting that providers were more aware of the need to refer patients and more confident that patients would complete referrals. Our experience underscores how the STRENGTHS implementation strategy aligned with broader health system reform, a critical step to enhancing implementation fidelity and sustainability. Similarly, in addition to standard clinical training related to patient privacy and protected health information, peer navigators participating in the STRENGTHS study also will receive CITI Research training for ethics in clinical research.

Challenges that arose during the design process, rather than exposing weaknesses in the implementation strategy, prompted critical discussions that demonstrate the strengths of the participatory design methods. One example was the persistent concern regarding prohibitive referral-related costs. Providing funds or travel vouchers was felt to be outside the scope of the study. However, we found that peer navigators could elucidate the potential daily-life impacts, disability, and catastrophic healthcare expenses associated with complications of uncontrolled hypertension. These counseling strategies demystified what is colloquially known as a ‘silent killer’ and reinforced the benefits of referral completion, which were especially powerful when conveyed from a peer (i.e. fellow patient) perspective [35].

**Fig. 3:**
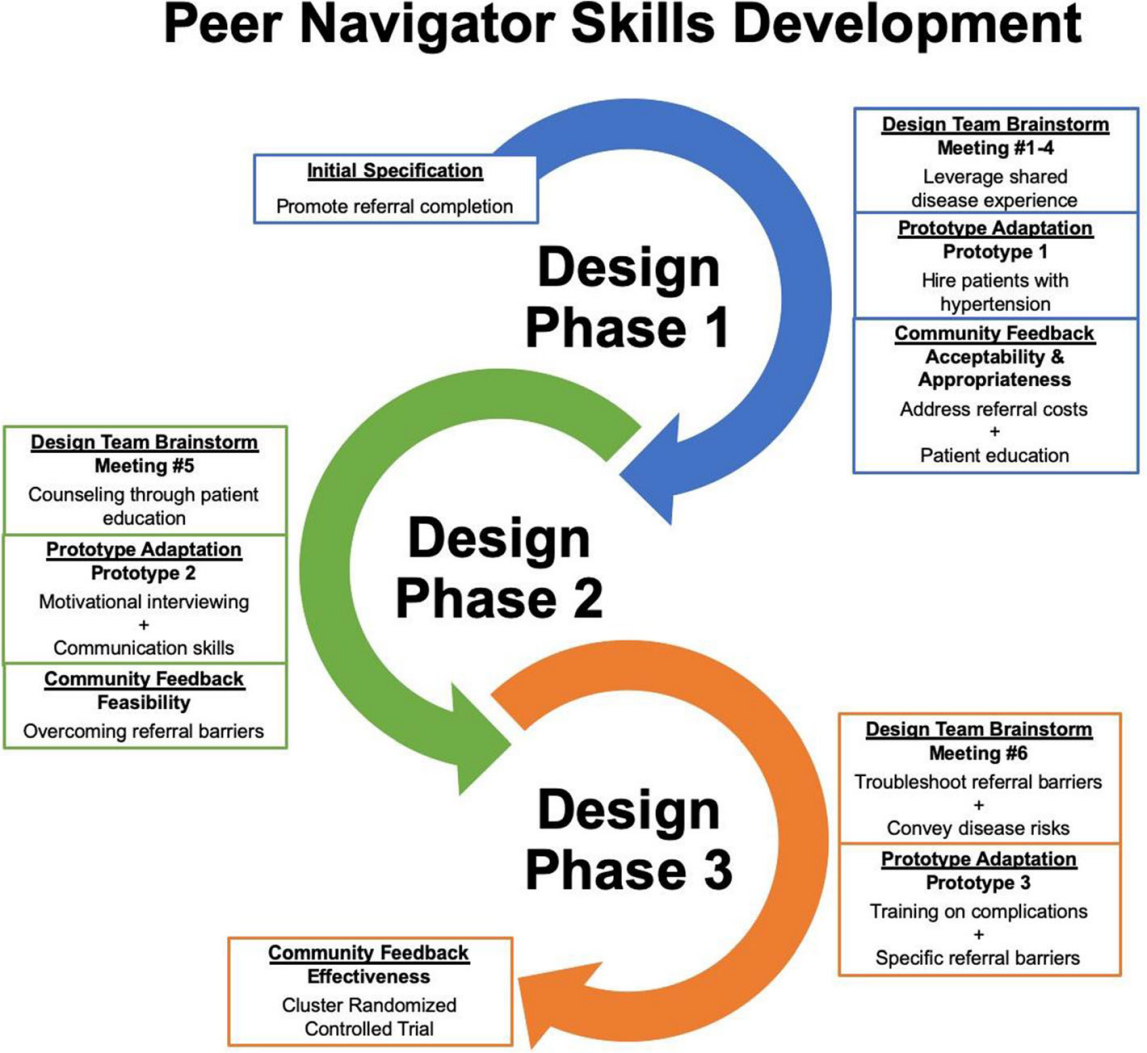
Example of Iterative Redesign during STRENGTHS Design Process – Peer Navigator Skills Development

Figure 3 demonstrates iterative redesign of key peer navigator skills to address this challenge. In prototype 1, peer navigators promoted referral completion through shared disease experience, but lacked communication skills and ways to address patients’ cost-related concerns. Prototype 2 addressed these deficits by adding peer navigator training in motivation interviewing and education on hypertension. However, feedback from feasibility testing indicated that addressing these persistent referral barriers required more active counseling strategies. As a result, prototype 3 equipped peer navigators with additional tools to address specific referral barriers (e.g. possible ways to mitigate transportation costs) and emphasize the risks of uncontrolled blood pressure. This trajectory illustrates not only how the design process added contextually specific details to initial specifications, but also how community feedback drove iterative redesign to enhance implementation.

### Advancing human-centered implementation research

**Fig. 4:**
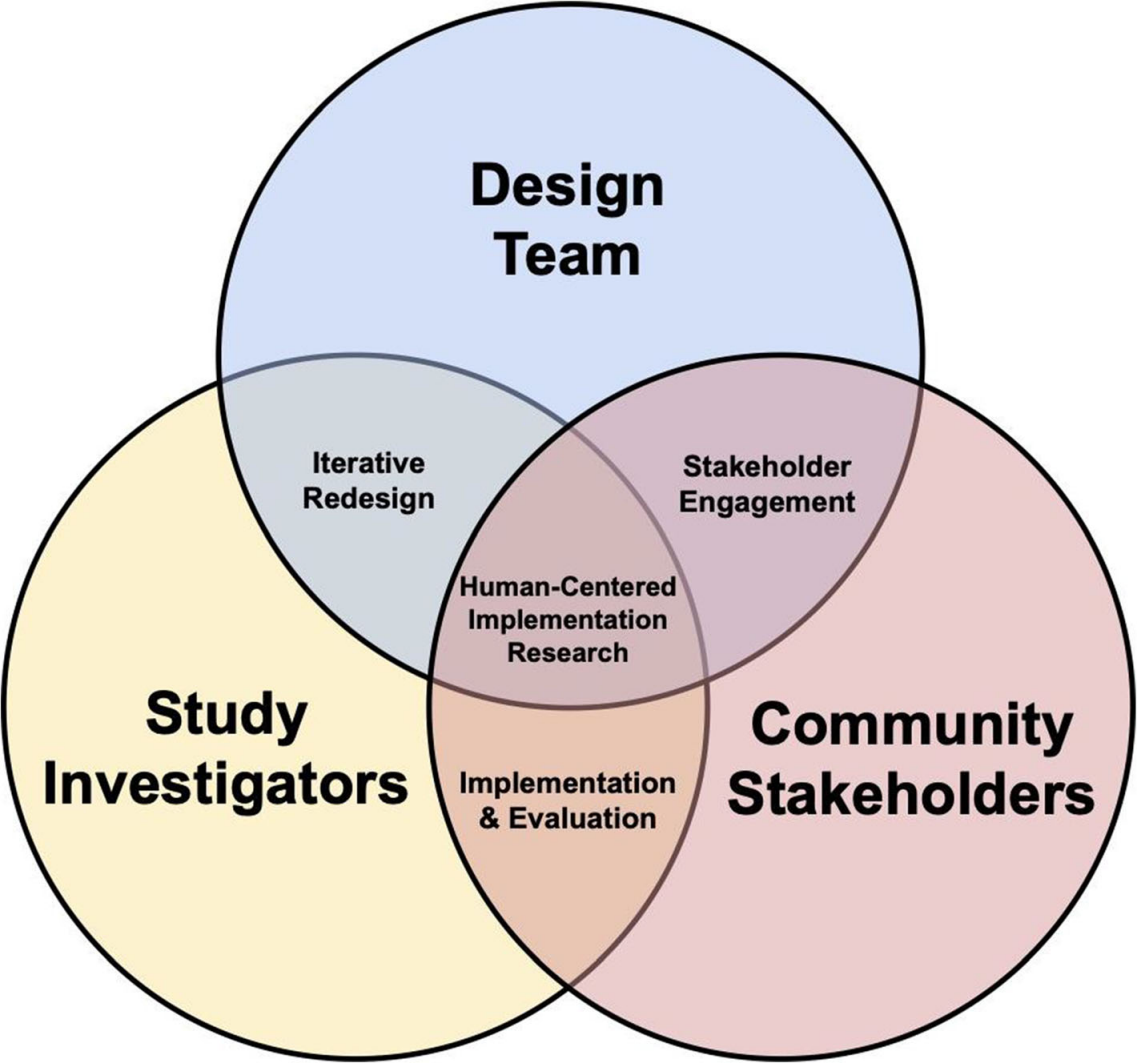
Conceptual Model for Human-Centered Implementation Research – an approach to strengthening partnerships between community stakeholders, the design team, and study investigators by defining their collaboration in core activities of implementation science: iterative and contextually-driven redesign, structured stakeholder engagement, and cycles of implementation and evaluation in the community

Our approach builds on the existing literature for design-focused implementation by integrating participatory design principles into the pre-implementation stage of the STRENGTHS study [36]. Based on our experience, we propose “human-centered implementation research” as a new conceptual framework that builds on the extensive base of stakeholder engagement literature, and defines inter-linkages and partnerships among key actors (community stakeholders, the design team, and study investigators) in the core activities of HCD and implementation science: iterative and contextually driven redesign, structured stakeholder engagement, and cycles of implementation and evaluation in the community (Fig. 4).

The STRENGTHS design process enhanced *stakeholder engagement* by including community members (including local clinicians and administrators) on the design team, which additionally reviewed and interpreted information gathered during the baseline needs and contextual assessment. Early and frequent stakeholder engagement ensured consistent alignment with health system initiatives and increased capacity for design thinking in the community [26].

*Iterative redesign* enabled collaboration between the design team and study investigators, who provided an additional layer of input on design products from the HCD process. Our design team used a co-creation approach, in which participants represented end-users of the implementation strategy (not an external design thinking team). As has been described in prior studies in this setting, co-creation adds contextual insights not only to design products but also to implementation processes by building partnerships with local stakeholders [18, 37].

Through *implementation and evaluation* of successive prototypes, we generated actionable feedback from community stakeholders. This approach introduced pragmatic boundaries to the HCD process to better align with implementation research methods [13]. Acceptability, appropriateness, and feasibility testing—core concepts of the implementation science literature—formalized iterative feedback cycles of HCD. The final prototype—the ultimate product of the HCD process—will be implemented and evaluated in the cluster-randomized controlled trial. We will therefore be able to evaluate whether our approach and method for integrating peer support strategies and HIT can address gaps in referral systems for chronic disease management.

Human-centered implementation research seeks to address a central tension in applying a design thinking mindset to implementation research: how to integrate HCD practices such as dynamic iteration, tolerance for ambiguity, and rapid prototyping, with aspects of traditional research where the study plans and procedures are often determined a priori [13]. As we found in STRENGTHS, the original project scope occasionally constrained the design team’s brainstorming activities, and some design team participants remained uncertain about how the design process would ultimately inform the implemented product. We recognize that addressing these challenging questions—regarding transparency of the design process, use of pre-determined intervention specifications in the study protocol, and relationship between investigators and design team members—remain central to successful human-centered implementation research.

Nevertheless, our experience with STRENGTHS suggests that integrating HCD methods with implementation research—rather than manifesting an “inherent tension” between these disciplines—may optimize implementation strategies initially proposed by study investigators [13]. Design team meetings enhanced scaffold specifications described in the study protocol (i.e. use of peers and HIT) through culturally and contextually specific details (e.g. defining the peer navigator role, determining appropriate training, designing the HIT interface). These essential details prioritized community perspectives, reflecting how human-centered implementation research may harness the creativity of HCD within the adaptation-focused framework of implementation science. We anticipate and hope that our approach may be replicable in other settings.

#### Limitations

We recognize that the ultimate product of our human-centered implementation research process was specific to our context in western Kenya and therefore may lack generalizability. However, we feel that our overall approach—human-centered implementation research—can be relevant for other settings. We acknowledge that our assessment of design team members’ reflections was limited in scope, but we feel that important insights were generated that will inform future studies and HCD implementation. We also recognize that appropriateness (not volume) of referrals is an important outcome to assess; due to the small number of participants in the feasibility pilot that was conducted, there were insufficient data to draw broader conclusions about the intervention’s ability to prevent avert unnecessary referrals. This important referral process metric is being actively assessed in the ongoing STRENGTHS trial. Finally, as the cluster-randomized controlled trial studying the STRENGTHS implementation strategy is ongoing, we cannot determine the ultimate effectiveness in terms of referral process metrics or health outcomes.

## Conclusion

In this study, we utilized a human-centered implementation research process to design the implementation strategy prototypes and test them for acceptability, appropriateness, and feasibility. Our experience supports the use of human-centered implementation research to create contextually specific implementation strategies. Our new conceptual model supplements previous literature to more fully capture the relationship between HCD and implementation research by emphasizing inter-linkages among study investigators, design team members, and community stakeholders. This approach may be used in different settings to enhance stakeholder engagement, implementation research, and population health.

## Supplementary Information

**Additional file 1.**

**Additional file 2.**

**Additional file 3.**

## Data Availability

The following items have been attached to this submission as appendices: • Focus group discussion guide for acceptability and appropriateness testing • Focus group discussion guide for feasibility testing • Focus group discussion guide for design team feedback The datasets used and/or analysed during the current study are available from the corresponding author on reasonable request.

## Abbreviations

STRENGTHS: Strengthening Referral Networks for Management of Hypertension Across the Health System
AMPATH: Academic Model Providing Access to Healthcare
CDM: Chronic Disease Management Program
AMRS: AMPATH Medical Record System
