# Supplementary material for: Human-centered implementation research: a new approach to develop and evaluate implementation strategies for strengthening referral networks for hypertension in western Kenya
Source: BMC Health Serv Res. 2021 Sep 3;21:910. doi: 10.1186/s12913-021-06930-2 (PMC8414706; doi:10.1186/s12913-021-06930-2)
Supplement: Supplementary file 1 — Additional file 1. [file 12913_2021_6930_MOESM1_ESM.docx]

**FGD Guide for Pilot Testing with Patients**

**Background:** Welcome to our group discussion. Hypertension is a major risk factor for cardiovascular disease and lack of coordination between different levels of the health system threaten the ability to provide the care necessary to control hypertension. Strong referral networks have improved health outcomes for chronic disease in a variety of settings.

The goal of the STRENGTHS study is to improve patient referrals using peer support and health information technology. During a preliminary phase of the study, we sought feedback from the community and identified the following barriers to referral:

- The challenge of navigating new, unfamiliar health facilities
- The lack of health education and psychosocial support
- Inadequate provider follow-up to encourage referral completion
- Inefficient sharing of health information between providers

To address these issues, we developed a peer navigator program using a design team of patients, providers, administrators, and Health Information Technology experts. This intervention included:

- Individualized peer support (peer navigator) to provide follow up, health education, and logistical support for patients
- A referral record system to track patient referral completion and prompt follow up
- Standardized referral documentation to help share information between providers

You have participated in a pilot study which tests our combined electronic referral record system and peer navigator program. We value your feedback on your experience during this pilot study so that we can improve our intervention. After integrating your feedback, we will be testing the intervention in a larger group of patients over the next year. This is why we have asked you to participate in our discussion today.

**FGD Rules**

1. There are no right or wrong answers. We expect that you will have differing points of view. Please share your point of view even if it differs from what others have said.
2. We are recording this session as we discussed, because we do not want to miss any of your comments. No names will be included in any reports. Your comments are confidential.
3. Don’t feel like you have to respond all the time. But if you want to follow up on something that someone has said, agree, disagree, or give an example, feel free to do that.
4. I am here to ask questions, listen, and make sure everyone has a chance to share. We’re interested in hearing from each of you. So if you are talking a lot, I may ask you to give others a chance. And if you are not saying much, I may call on you. We just want to make sure all of you have a chance to share your ideas.
5. If you have a cell phone, please put it on silent mode.

**I. Feasibility of the STRENGTHS Intervention**

- What was your experience with the peer navigator program?
- Can you describe a typical visit with your peer navigator, step-by-step?
  - *Probe: How would you communicate?*
  - *Probe: How often would you meet?*
  - *Probe: Where would you meet?*
- What specifically did you discuss during your meetings?
  - *Probe: Did your peer navigator provide psychosocial support? What was effective about how your peer navigator did this? What could have been done differently?*
  - *Probe: Did your peer navigator help you navigate new health facilities or help you travel to your appointments? What was effective about how your peer navigator did this? What could have been done differently?*
  - *Probe: Did your peer navigator help remind you to attend appointments? What was effective about how your peer navigator did this? What could have been done differently?*
  - *Probe: Is there anything else you wish your peer navigator had done for you or discussed with you?*
- What would you change about the meetings with your peer navigator?
- How did you feel about sharing personal health information with your peer navigator?
  - *Probe: Did you have any concerns with sharing your health information?*
  - *Probe: Was your peer navigator punctual? Respectful? Organized?*
- How was your experience being referred from a facility?
  - *Probe: Was the reason for referral explained to you clearly?*
  - *Probe: Did the referring facility offer any contact information for the new facility or assistance in traveling there?*
  - *Probe: Did the referring facility or provider connect you with the peer navigator?*
- How was your experience being received into a new facility during your referral appointment?
  - *Probe: Did the provider at the receiving facility have your health information or a record of your referral?*
  - *Probe: Were the plans for clinic follow up explained to you clearly?*
  - *Probe: Did you communicate with your peer navigator after the visit? If so, what did you discuss with them?*
- What would you change about the referral process and clinical record system?

**II. Conclusion and Wrap Up**

- Is there anything that we should have talked about on this matter but have not yet discussed?
- Thank you for your time and participation. We very much appreciate your comments, discussion, and input. We plan to take into account everything that was said today as we continue to improve the services we offer to your community.

.
