# Supplementary material for: Human-centered implementation research: a new approach to develop and evaluate implementation strategies for strengthening referral networks for hypertension in western Kenya
Source: BMC Health Serv Res. 2021 Sep 3;21:910. doi: 10.1186/s12913-021-06930-2 (PMC8414706; doi:10.1186/s12913-021-06930-2)
Supplement: Supplementary file 2 — Additional file 2. [file 12913_2021_6930_MOESM2_ESM.docx]

**FGD Guide for Pilot Testing with Providers/Admin**

**Background:** Welcome to our group discussion. Hypertension is a major risk factor for cardiovascular disease and lack of coordination between different levels of the health system threaten the ability to provide the care necessary to control hypertension. Strong referral networks have improved health outcomes for chronic disease in a variety of settings.

The goal of the STRENGTHS study is to improve patient referrals using peer support and health information technology. During a preliminary phase of the study, we sought feedback from the community and identified the following barriers to referral:

- The challenge of navigating new, unfamiliar health facilities
- The lack of health education and psychosocial support
- Inadequate provider follow-up to encourage referral completion
- Inefficient sharing of health information between providers

To address these issues, we developed a peer navigator program using a design team of patients, providers, administrators, and Health Information Technology experts. This intervention included:

- Individualized peer support (peer navigator) to provide follow up, health education, and logistical support for patients
- A referral record system to track patient referral completion and prompt follow up
- Standardized referral documentation to help share information between providers

You have participated in a pilot study, which tests our combined electronic referral record system and peer navigator program. We value your feedback on your experience during this pilot study so that we can improve our intervention. After integrating your feedback, we will be testing the intervention in a larger group of patients over the next year. This is why we have asked you to participate in our discussion today.

**FGD Rules**

1. There are no right or wrong answers. We expect that you will have differing points of view. Please share your point of view even if it differs from what others have said.
2. We are recording this session as we discussed, because we do not want to miss any of your comments. No names will be included in any reports. Your comments are confidential.
3. Don’t feel like you have to respond all the time. But if you want to follow up on something that someone has said, agree, disagree, or give an example, feel free to do that.
4. I am here to ask questions, listen, and make sure everyone has a chance to share. We’re interested in hearing from each of you. So if you are talking a lot, I may ask you to give others a chance. And if you are not saying much, I may call on you. We just want to make sure all of you have a chance to share your ideas.
5. If you have a cell phone, please put it on silent mode.

**I. Key Questions**

***Feasibility of the STRENGTHS Intervention***

**A. For Providers**

- What was your experience with the combined electronic referral record system and peer navigator program?
- How did you communicate with peer navigators regarding patient referrals?
- What other interactions did you have with peer navigators? Please describe these interactions.
- What is your opinion on the characteristics of peer navigators in the pilot?
  - *Probe: Did you feel that they were professional, respectful of patient confidentiality, and integrate well with the clinical environment?*
- What would you change about the peer navigator program?
- What is your opinion on the effect of peer navigators on patient behavior?
  - *Probe: After meeting with peer navigators, did patients seem informed about their reason for referral?*
  - *Probe: After meeting with peer navigators, did patients still experience barriers to completing referrals? If so, what were these barriers?*
- What is your opinion on the referral record forms?
  - *Probe: Were there times when you didn’t use it? What factors contributed to its use or nonuse?*
  - *Probe: Did they convey up-to-date information regarding your patient referrals?*
- What is your opinion on data forms used by peer navigators?
  - *Probe: What did you think about the peer navigator encounter forms? Were these helpful?*
  - *Probe: What did you think about the peer navigator communication function? Did you use this to discuss patient referrals with peer navigators?*
- What is the clinical record system used at your facility?
  - *Probe: Does your facility use paper records or an EMR (e.g. Muzima or POC)?*
  - *Probe: How did the referral forms integrate with your other clinical record keeping systems?*
  - *Probe: What did you think about the decision support function?*
  - *Probe: Do you feel that this referral record system helped coordinate patient referrals with your colleagues at other facilities?*
  - *Probe: What would you change about the referral record forms used at facilities?*

**B. For Administrators**

- What was your experience with the combined electronic referral record system and peer navigator program?
- How did you communicate with peer navigators regarding patient referrals?
- What other interactions did you have with peer navigators? Please describe these interactions.
- What is your opinion on the characteristics of peer navigators in the pilot?
  - *Probe: Did you feel that they were professional, respectful of patient confidentiality, and integrate well with the clinical environment?*
- What would you change about the peer navigator program?
- What is your opinion on the effect of peer navigators on patient behavior?
  - *Probe: After meeting with peer navigators, did patients seem informed about their reason for referral?*
  - *Probe: After meeting with peer navigators, did patients still experience barriers to completing referrals? If so, what were these barriers?*
- What is your opinion on the referral record forms?
  - *Probe: Were there times when you didn’t use it? What factors contributed to its use or nonuse?*
  - *Probe: Did they convey up-to-date information regarding your patient referrals?*
- What is your opinion on data forms used by peer navigators?
  - *Probe: What did you think about the peer navigator encounter forms? Were these helpful?*
  - *Probe: What did you think about the peer navigator communication function? Did you use this to discuss patient referrals with peer navigators?*
- What is the clinical record system used at your facility?
  - *Probe: Does your facility use paper records or an EMR (e.g. Muzima or POC)?*
  - *Probe: How did the referral forms integrate with your other clinical record keeping systems?*
  - *Probe: What did you think about the decision support function?*
  - *Probe: Do you feel that this referral record system helped coordinate patient referrals with your colleagues at other facilities?*
  - *Probe: What would you change about the referral record forms used at facilities?*
- Describe your experience using AMRS to track clinic data for patient referrals.
  - *Probe: How were you able to monitor patient referrals?*
  - *Probe: How were you able to identify patients with incomplete referrals or loss-to-follow-up? Was this information shared with the appropriate peer navigator?*
  - *Probe: How were you able to access referral records generated at clinics? For paper records, how were these entered into AMRS? How long did it take from the date of appointment to date of entry?*
  - *Probe: How were you able to communicate referral records to sending or receiving clinics?*
  - *Probe: What were the gaps in referral data collection?*
  - *Probe: What would you change about the referral record system?*

**II. Conclusion and Wrap Up**

- Is there anything that we should have talked about on this matter but have not yet discussed?
- Thank you for your time and participation. We very much appreciate your comments, discussion, and input. We plan to take into account everything that was said today as we continue to improve the services we offer to your community.
