# Supplementary material for: Human-centered implementation research: a new approach to develop and evaluate implementation strategies for strengthening referral networks for hypertension in western Kenya
Source: BMC Health Serv Res. 2021 Sep 3;21:910. doi: 10.1186/s12913-021-06930-2 (PMC8414706; doi:10.1186/s12913-021-06930-2)
Supplement: Supplementary file 3 — Additional file 3. [file 12913_2021_6930_MOESM3_ESM.docx]

**SEMI STRUCTURED INTERVIEW PILOT GUIDE FOR PEER NAVIGATORS**

**STUDY:** **Strengthening Referral Networks for Management of Hypertension across the Health System (STRENGTHS)**

**Cover Sheet**

**Interviewer: _________________________________________**

**Date: ____/____/___________**

**Time: ______________________________________________**

**Location/Venue: ____________________________________**

**SII Completion Check List:**

**( ) Participant’s informed consent fully documented?**

**( ) Study cover sheet attached to the SII field notes?**

**********************************************************

**Semi structured Interview Guide**

**Background:** Welcome to our group discussion. Hypertension is a major risk factor for cardiovascular disease and lack of coordination between different levels of the health system threaten the ability to provide the care necessary to control hypertension. Strong referral networks have improved health outcomes for chronic disease in a variety of settings.

The goal of the STRENGTHS study is to improve patient referrals using peer support and health information technology. During a preliminary phase of the study, we sought feedback from the community and identified the following barriers to referral:

- The challenge of navigating new, unfamiliar health facilities
- The lack of health education and psychosocial support
- Inadequate provider follow-up to encourage referral completion
- Inefficient sharing of health information between providers

To address these issues, we developed a peer navigator program using a design team of patients, providers, administrators, and Health Information Technology experts. This intervention included:

- Individualized peer support (peer navigator) to provide follow up, health education, and logistical support for patients
- A referral record system to track patient referral completion and prompt follow up
- Standardized referral documentation to help share information between providers

You have participated in a pilot study, which tests our combined electronic referral record system and peer navigator program. We value your feedback on your experience during this pilot study so that we can improve our intervention. After integrating your feedback, we will be testing the intervention in a larger group of patients over the next year. This is why we have asked you to participate in our discussion today.

As we start our interview, please put your cellphone on silent mode.

**I. Key Questions**

**Feasibility of the STRENGTHS intervention**

- What was your experience with the combined electronic referral record system and peer navigator program?
- How did you communicate with other peer navigators regarding patient referrals?
- How did you communicate with other research team i.e Research Assistants regarding patient referrals?
- How did you communicate with the providers and other administrator’s regarding patient referrals?
- What other interactions did you have with other following people? Please describe these interactions.

*Probes: Other peer navigators?*

*Probes: Providers?*

*Probes: Research team?*

*Probes: Patients*

- What is your opinion on the characteristics of peer navigators in the pilot?
  - *Probe: Did you feel that they were professional, respectful of patient confidentiality, and integrate well with the clinical environment?*
- What would you change about the peer navigator program?
- What is your opinion on the effect of peer navigators on patient behavior?
  - *Probe: After meeting with peer navigators, did patients seem informed about their reason for referral?*
  - *Probe: After meeting with peer navigators, did patients still experience barriers to completing referrals? If so, what were these barriers?*
- What is your opinion on the referral record forms?
  - *Probe: Were there times when you didn’t use it? What factors contributed to its use or nonuse?*
  - *Probe: Did they convey up-to-date information regarding your patient referrals?*
- What is your opinion on data forms used by peer navigators?
  - *Probe: What did you think about the peer navigator encounter forms? Were these helpful?*
  - *Probe: What did you think about the peer navigator communication function? Did you use this to discuss patient referrals with peer navigators?*
- What is the clinical record system used at your facility?
  - *Probe: Does your facility use paper records or an EMR (e.g. Muzima or POC)?*
  - *Probe: How did the referral forms integrate with your other clinical record keeping systems?*
  - *Probe: What did you think about the decision support function?*
  - *Probe: Do you feel that this referral record system helped coordinate patient referrals with your colleagues at other facilities?*
  - *Probe: What would you change about the referral record forms used at facilities?*

**II. Conclusion and Wrap Up**

- Is there anything that we should have talked about on this matter but have not yet discussed?
- Thank you for your time and participation. We very much appreciate your comments, discussion, and input. We plan to take into account everything that was said today as we continue to improve the services we offer to your community.
